# Effects of MRI radiomics combined with clinical data in evaluating lymph node metastasis in mrT1-3a staging rectal cancer

**DOI:** 10.3389/fonc.2023.1194120

**Published:** 2023-10-16

**Authors:** Xue Dong, Gang Ren, Yanhong Chen, Huifang Yong, Tingting Zhang, Qiufeng Yin, Zhongyang Zhang, Shijun Yuan, Yaqiong Ge, Shaofeng Duan, Huanhuan Liu, Dengbin Wang

**Affiliations:** ^1^Department of Radiology, Xinhua Hospital, Shanghai Jiao Tong University School of Medicine, Shanghai, China; ^2^Department of Radiology, Integrated Traditional Chinese and Western Medicine Hospital, Shanghai, China; ^3^Department of Medicine, GE Healthcare China, Shanghai, China

**Keywords:** rectal cancer, magnetic resonance imaging, radiomics, lymph node, metastasis

## Abstract

**Objective:**

To investigate the value of a clinical-MRI radiomics model based on clinical characteristics and T2-weighted imaging (T2WI) for preoperatively evaluating lymph node (LN) metastasis in patients with MRI-predicted low tumor (T) staging rectal cancer (mrT1, mrT2, and mrT3a with extramural spread ≤ 5 mm).

**Methods:**

This retrospective study enrolled 303 patients with low T-staging rectal cancer (training cohort, n = 213, testing cohort n = 90). A total of 960 radiomics features were extracted from T2WI. Minimum redundancy and maximum relevance (mRMR) and support vector machine were performed to select the best performed radiomics features for predicting LN metastasis. Multivariate logistic regression analysis was then used to construct the clinical and clinical-radiomics combined models. The model performance for predicting LN metastasis was assessed by receiver operator characteristic curve (ROC) and clinical utility implementing a nomogram and decision curve analysis (DCA). The predictive performance for LN metastasis was also compared between the combined model and human readers (2 seniors).

**Results:**

Fourteen radiomics features and 2 clinical characteristics were selected for predicting LN metastasis. In the testing cohort, a higher positive predictive value of 75.9% for the combined model was achieved than those of the clinical model (44.8%) and two readers (reader 1: 54.9%, reader 2: 56.3%) in identifying LN metastasis. The interobserver agreement between 2 readers was moderate with a kappa value of 0.416. A clinical-radiomics nomogram and decision curve analysis demonstrated that the combined model was clinically useful.

**Conclusion:**

T2WI-based radiomics combined with clinical data could improve the efficacy in noninvasively evaluating LN metastasis for the low T-staging rectal cancer and aid in tailoring treatment strategies.

## Introduction

Colorectal cancer (CRC) is the fourth most commonly diagnosed cancer and second leading cause of cancer death worldwide, and rectal cancer accounts for approximately 40% of all CRC cases (1). In patients with early rectal cancer, the rate of lymph node (LN) metastasis is 12.2%-18.0% (2). LN staging is critical for therapeutic decision-making and the prognosis prediction in patients with rectal cancer. According to the 8th edition of the American Joint Committee on Cancer (AJCC) staging system and the National Comprehensive Cancer Network (NCCN) Clinical Practice Guidelines for rectal cancer (3–5), all patients with cT1N0M0 disease can directly receive transanal endoscopic microsurgery, while those with cT2N0M0 disease can be recommended to undergo total mesolectal excision. The AJCC recommended an optional stratification of T3 tumors according to the extramural depth of invasion: less than 5 mm, T3a; 5-10 mm, T3b; and more than 10 mm, T3c (3). The extramural depth of invasion of less than 5 mm (T3aN0M0) may be adequately managed with surgery alone and have a prognosis comparable to that of tumors characterized as “T1/T2” (5). However, if LN metastasis exists in the patients with T1-3a staging (cT1-3aN1-2M0), it is strongly suggested that patients receive neoadjuvant chemoradiotherapy (nCRT); otherwise, the recurrence rate would increase (6). By contrast, unnecessary nCRT may be performed if LN staging is overestimated, resulting in potential complications such as dysuria and sexual dysfunction (7, 8). Therefore, accurate preoperative assessment of LN staging is the premise for precision treatment in T1-3a rectal cancers, and also directly affects the prognosis in patients with rectal cancer.

Magnetic resonance imaging (MRI) has been considered as the modality of choice due to its excellent soft tissue resolution for preoperative local staging of rectal cancer (4). However, the pre-treatment diagnosis of LN metastasis remains clinically challenging. The most common criteria are the traditional morphological findings, such as short-axis diameter, shape, border, and signal intensity (9). Previous studies have revealed that MRI can achieve sensitivities of 58%-70% and specificities of 75%-85% by using the short axial diameter criterion for evaluating LN metastasis (10), while its application is limited since LN enlargement can be also caused by inflammation or reactive hyperplasia. Morphological features, such as nodal borders or an internal signal pattern, are reported to be potential predictive factors of LN metastasis (11, 12), however, their predictive value remains controversial due to observer dependence (13).

Radiomics, which involve high-throughput extraction of a large number of quantitative features from medical images, has attracted an increasing attention in recent years. MRI-based radiomics has been proved to improve the predictive accuracy of tumor characteristics, phenotypic subtype classification, prognosis, or treatment response compared with conventional MRI (14–16). With regard to rectal cancer, prior studies about predicting LN metastasis based on MRI radiomics focused on advanced rectal cancer (17–19). So far, to our best knowledge, few studies have investigated the value of radiomics in predicting LN metastasis in low T-staging (T1-3a) rectal cancer.

In the present study, we aim to assess the value of MRI radiomics based on T2-weighted imaging (T2WI) for the individualized preoperative evaluation of LN metastasis in low T-staging rectal cancer patients, and to develop a clinical-radiomics combined model that could aid in improving decision-making and guiding individualized treatment.

## Materials and methods

This retrospective study was approved by the Institutional Ethics Committee of *BLINDED*, and the informed consent requirement was waived for this retrospective study using de-identified data.

### Patients

**Figure 1:**
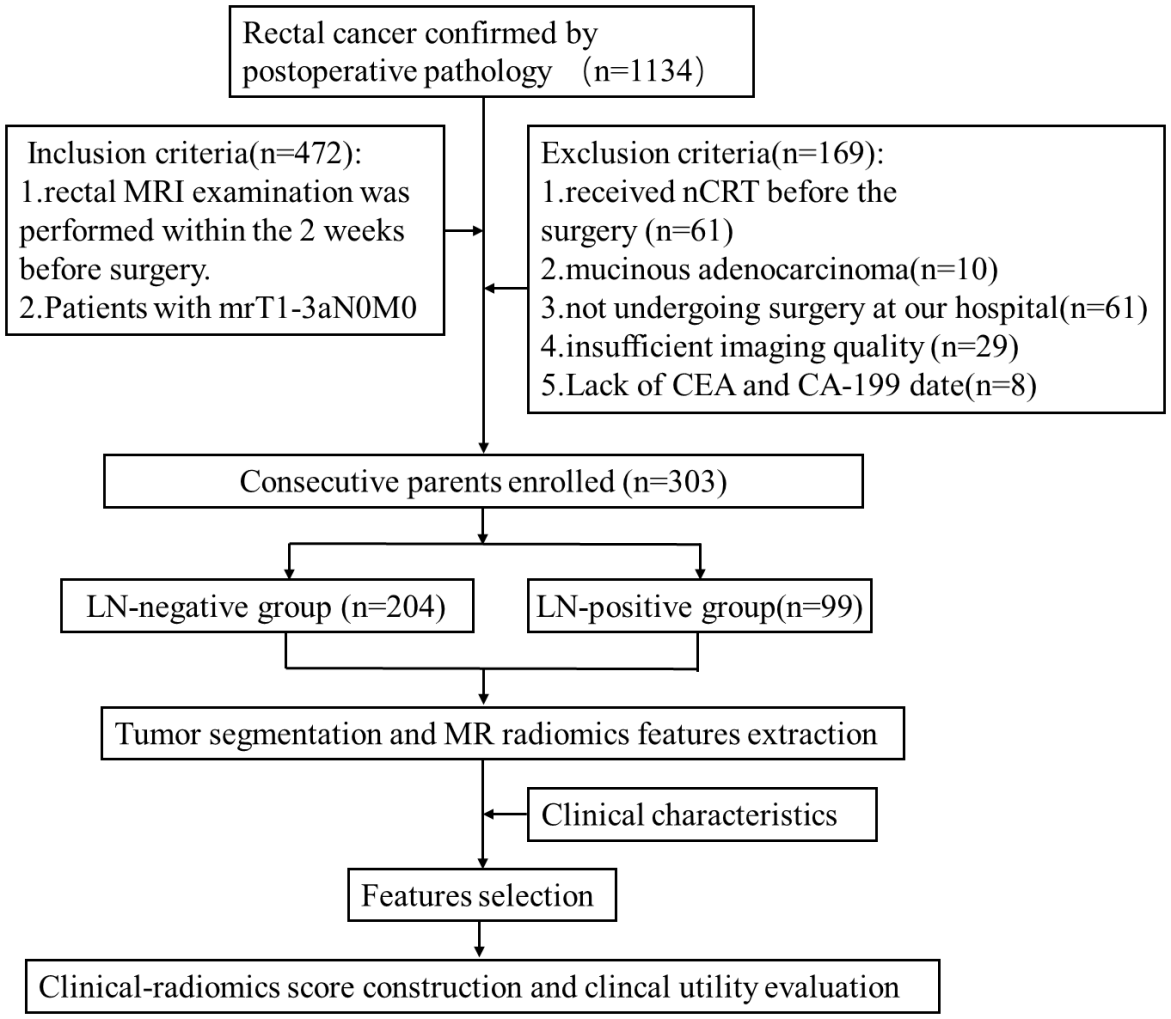
Workflow of this study.

Data of 1134 patients with rectal adenocarcinoma from October 2015 to July 2021 confirmed by postoperative pathology in our institution were reviewed in this retrospective study. Baseline clinicopathologic data, including gender, age, pretreatment carcinoembryonic antigen (CEA) level, carbohydrate antigen 199 (CA199) level, and pathological LN status were collected. In addition, the MRI features including tumor location, tumor length, MR-predicted tumor (mrT) staging, MR-predicted LN (mrN) staging, and distant metastasis were obtained. The patients who underwent rectal MRI within 2 weeks before surgery and were staged as mrT1-3aN0M0 were initially recruited. Totally 169 patients were excluded according to the criteria shown in **Figure 1**. Finally, 303 patients (mean age,64 years; range, 28 to 90 years) were enrolled in this study. According to the pathological reports, there were 99 patients with LN metastasis, defined as positive LN metastasis (LN+), and 204 patients without LN metastasis defined as negative lymph node involvement (LN-). The patients were randomly divided into the training (n = 213) and testing (n = 90) cohorts in a ratio of 7:3. The workflow of this study is shown in **Figure 1**.

### Image acquisition

All high-resolution rectal MRI were performed on a 3.0T MR scanner (Ingenia, Philips Medical Systems) using a 32-channel phase-array body coil with patients in the supine position. The standard rectal MRI protocols including sagittal T2WI, oblique axial T2WI, coronal T2WI, and diffusion weighted imaging (DWI) with two b-factors (0 and 1000 s/mm^2^) sequences were conducted. The oblique axial T2WI sequence was determined in the sagittal position, which was perpendicular to the long axis of the rectal tumor according to the following parameters: a field of view of 180 mm × 180 mm, a repetition time of 3,500 ms, an echo time of 100 ms, an echo train length of 24, a thickness of 3.0 mm, and a gap of 0.3 mm.

### Image evaluation

Two radiologists (*BLINDED*, with 5 and 10 years of experience in gastrointestinal imaging, respectively) independently measured tumor length based on sagittal T2WI sequence. Three radiologists (*BLINDED*, with 3,10, and 33 years of experience in gastrointestinal imaging, respectively) independently reviewed the pretreatment MR images for assessing the tumor location and mrT staging. All the radiologists were blinded to the histopathology results. MRI features were evaluated according to the following criteria: (1) mrT staging: high resolution T2WI and DWI images are the main evaluation sequences. According to the AJCC and NCCN guidelines (8th edition) (3), Stage T1/T2: tumors were limited within muscularis propria, since differentiation of T1 from T2 tumors on MRI was difficult; Stage T3: the tumor penetrated the muscularis propria and reached the subserosal or mesorectum. T3 tumor was further categorized into the early T3 tumor (T3a, extramural spread ≤ 5 mm) and advanced T3 tumor (extramural spread > 5 mm) due to the different prognosis (3). Stage T4: tumors infiltrated the peritoneum or peritoneal reflection (T4a) or surrounding organs and structures (T4b). (2) For mrN staging: in reference to the recommendations of the European Society of Gastrointestinal and Abdominal Radiology (ESGAR) (9), metastatic LNs are defined as: a). short diameter ≥ 9 mm; b). The short diameter of 5 – 8 mm meets two morphological indexes at the same time; c) The short diameter < 5mm meets three morphological indexes at the same time; d) LNs with mucus signal (regardless of size). The morphological indexes of LN metastasis were round LNs, irregular edges and uneven internal signals. (3) Tumor length: the longest longitudinal diameter of the tumor in sagittal sequence. (4) Tumor location: it was measured on the approximate luminal center of the rectum on the sagittal T2WI sequence and categorized as low (0–5 cm), middle (5.1–10 cm), and high (10.1–15 cm) according to the distance from the anal verge to the lowest edge of the tumor (20).

The pathological LN status of each patient was recorded according to the postoperative histopathological reports.

### Image segmentation

**Figure 2:**
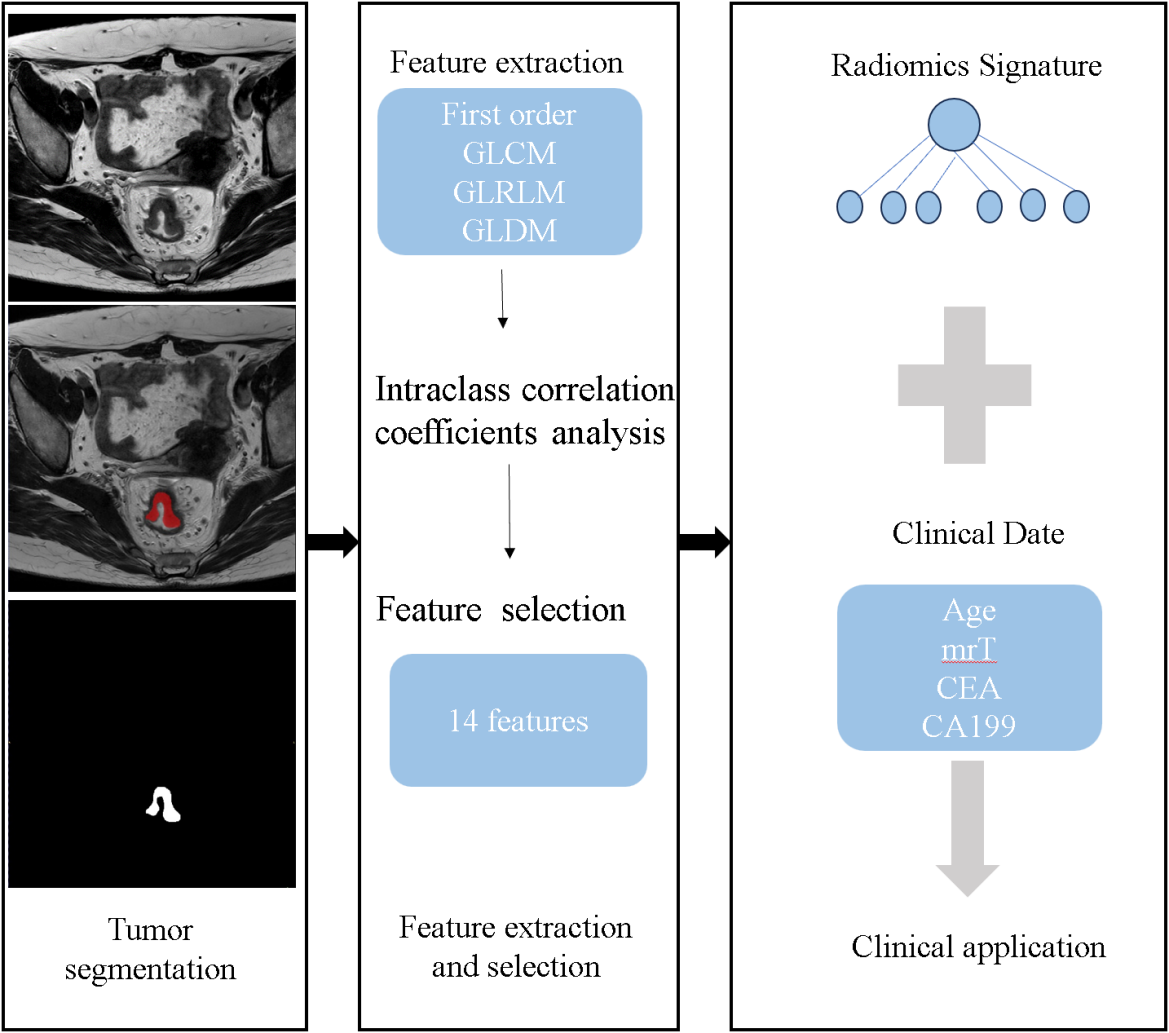
The framework for the radiomics workflow.

The ITK-SNAP software (version 3.8.0, www.itksnap.org), a free open-source software package, was employed for manual segmentation of the entire volume of three-dimensional (3D) volume of interest (VOI), including the whole tumor and excluding obvious necrosis, gas, hemorrhage and lumen content areas. One radiologist (*BLINDED*) who was blinded to the histopathology results manually segmented the contour of the tumor on oblique axial T2WI images, then, the corresponding VOI was automatically generated. The segmented VOIs were reviewed and modified by a radiologist (*BLINDED*, with 15 years of experience in abdominal MRI diagnosis) who was blinded to the histopathology results. The primary tumor was identified as areas of intermediate signal intensity on T2WI, high-signal intensity on DWI (b=1000 s/mm^2^), and corresponding low-signal intensity on apparent diffusion coefficient map. An overview of the radiomics analysis workflow is shown in **Figure 2**.

We randomly chose 20 VOIs for evaluating the inter- and intraobserver reproducibility of radiomics feature extraction. VOI segmentation was performed again by the same radiologist (*BLINDED*) a month later, as well as by another radiologist (*BLINDED*).

### Radiomics feature extraction and selection

Minimum redundancy and maximum relevance (mRMR) and support vector machine recursive feature elimination (SVM-RFE) were used to select the best performed radiomics features. A total of 960 radiomics features including first order statistics features, shape-based features, gray-level co-occurrence matrix (GLCM) features, gray-level run-length matrix (GLRLM) features, gray-level size zone matrix (GLSZM) features, and gray-level dependence matrix (GLDM) features were extracted from the oblique axial T2WI images.

### Development and validation of the clinical and clinical-radiomics combined models

For clinical model, univariate logistic regression analysis was first conducted with the following clinical-MRI information: age, gender, tumor length, tumor location, levels of CEA and CA199, and mrT staging to identify potential factors for evaluating metastatic LNs. Then multivariate logistic regression analysis was used to select the independent features. For the clinical-radiomics combined model, the selected radiomics and clinical features were included by using the multivariable logistic analysis via backward stepwise selection.

The performance of the clinical model and clinical-radiomics combined models in evaluating LN status was assessed in the training and testing cohorts by using the receiver operating characteristic curve (ROC) analysis and the areas under the curve (AUC). The corresponding specificity, sensitivity, negative predictive values (NPV), and positive predictive values (PPV) were then established. Decision curve analysis (DCA) was performed to determine the net benefits of the evaluation models at different threshold probabilities in the training cohort.

### Comparison of the performance between the clinical-radiomics model and radiologists for evaluating LN status

The testing cohort was used to compare the performance of the clinical-radiomics model with that of 2 senior radiologists (*BLINDED*, with 10 and 15 years of experience in gastrointestinal imaging, respectively) in evaluating LNs status. The 2 senior radiologists evaluated the LN status independently and were blinded to the pathological results.

### Statistical analysis

All statistical analyses were performed with SPSS 27.0 (IBM, New York, NY) and R software (version 3.4.2; Vienna, Austria, www.r-project.org). The model performance for predicting LN metastasis was assessed by receiver operator characteristic curve (ROC) and the area under curve (AUC). Delong’s test was used to compare the differences of AUCs for different models. Comparisons of patient characteristics between LN-negative and LN-positive groups were performed by independent two sample t-test, and Chi-squared or Fisher’s exact tests via SPSS 27.0. Other statistical analyses were performed with R software. *P* < 0.05 indicated statistically significant differences.

An intraclass correlation coefficients (ICC) was performed to evaluate the inter- and intra-observer agreements of radiomics feature extraction, and interobserver agreement for tumor length measurement between two readers, where an ICC of 0.81 to 1.00 showed almost perfect agreement, 0.61 to 0.80 as substantial agreement, and 0.41 to 0.60 as moderate agreement, 0.21 to 0.40 as a fair agreement, and 0 to 0.20 as a poor or no agreement (21). A Fleiss kappa coefficient was used for evaluating interobserver agreement among three readers regarding tumor location, mrT staging. Majority opinion was designated the final value. The kappa value was calculated to evaluate the diagnosis consistency between two radiologists for identifying LN metastasis, where the kappa value of 0.75 to 1.00 showed almost perfect consistency, 0.4 to 0.75 as moderate consistency, and 0 to 0.40 as a poor or no consistency (22).

## Results

### Patient characteristics

The detailed characteristics of patients in the training and testing cohorts are summarized in **Table 1**. There was no significant difference between two cohorts for LN metastasis occurrence (48.9% [70/213] and 32.2% [29/61] in the training and testing cohorts). mrT staging was significantly different between the LN-positive and LN-negative groups in the training cohort (*P* = 0.007). There were no significant differences between the LN-positive and LN-negative groups in terms of gender, age, tumor location, tumor length, CEA level, and CA199 level.

**Table 1:** Characteristics of patients in the training and testing cohorts.

Characteristics	Training cohort		*P*	Testing cohort		*P*
	pLN+	pLN-		pLN+	pLN-	
	(n=70)	(n=143)		(n=29)	(n=61)	
Gender (%)			0.324			0.108
Female	26(37.1)	42(29.4)		7(24.1)	27(44.3)	
Male	44(62.9)	101(70.6)		22(75.9)	34(55.7)	
Age, mean(SD)	64.6(11.3)	63.2(11.1)	0.395	61.5(10.8)	64.8(10.2)	0.163
CEA, ng/ml			0.069			0.338
< 10	50(71.4)	119(83.2)		21(72.4)	51(83.6)	
≥ 10	20(28.6)	24(16.8)		8(27.6)	10(16.4)	
CA199, U/ml			0.263			0.359
< 30	65(92.9)	139(97.2)		24(82.8)	56(91.8)	
≥ 30	5(7.1)	4(2.8)		5(17.2)	5(8.2)	
Tumor length (SD)	4.1(1.6)	3.9(1.2)	0.151	3.6(1.1)	3.7(1.4)	0.803
Tumor location			0.132			0.403
High	23(32.9)	29(20.3)		8(27.6)	21(34.4)	
Middle	20(28.6)	43(30.1)		13(44.8)	18(29.5)	
Lower	27(38.5)	71(49.6)		8(27.6)	22(36.1)	
mrT staging			0.007			0.427
T1/T2	36 (51.4)	72 (50.3)		18 (62.1)	32(52.5)	
T3a	34 (48.6)	71 (49.7)		11 (37.9)	29(47.5)	

pLN+, pathology-proved lymph node metastasis; pLN-, pathology-proved lymph node negative; CEA, carcinoembryonic antigen; CA199, carbohydrate antigen 199; sd, standard deviation. P < 0.05 is considered statistically significant.

There was good interobserver agreement between two readers in the measurements of tumor length (ICC = 0.989, 95% confidence interval [CI] = 0.986-0.991). Interobserver agreement among three readers was very good for tumor location (Fleiss’ kappa coefficient = 1.000, 95% CI = 1.000-1.000) and mrT staging (Fleiss’ kappa coefficient = 0.929, 95% CI = 0.881-0.976).

### Features selection, development, and validation of evaluation models

The interobserver and intra-observer reproducibility of radiomics feature extraction was satisfactory, and the ICCs of all extracted features were greater than 0.8.

Finally, 2 clinical and 14 radiomics features were selected after applying mRMR and SVM-RFE: CEA, mrT staging, wavelet_LLL_glcm_SumSquares, wavelet_LHL_ firstorder_RobustMeanAbsoluteDeviation, log_sigma_3_0_mm_3D_ glcm _ Cluster Prominence, wavelet_HLL_glszm_ZoneEntropy, wavelet_LLL_glcm_Id, log_sigma_ 5_0_mm_3D_glszm_HighGrayLevelZoneEmphasis, log_sigma_3_0_mm_3D_ first order_MeanAbsoluteDeviation, wavelet_HLL_glcm_SumSquares, log_sigma _3_0_mm_3D_gldm_HighGrayLevel Emphasis, wavelet_LHL_glcm_SumSquares, wavelet_LLH_gldm_ Dependence Variance, log_sigma_5_0_mm_3D_ glcm_ Joint Entropy, wavelet_HLL_glszm_High GrayLevelZoneEmphasis, wavelet_HLH_ gldm_HighGrayLevelEmphasis, wavelet_LLL_gldm_DependenceVariance.

**Figure 3:**
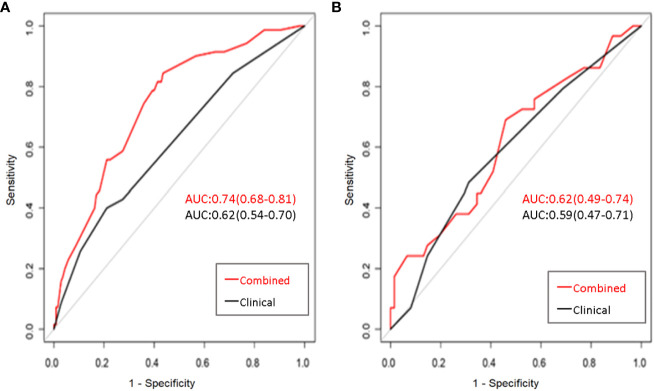
The receiver operator characteristic curves to identify the status of LNs for the clinical model and clinical-radiomics combined model in the training **(A)** and testing cohorts **(B)**. The predictive performance of the combined model for preoperative lymph node metastasis of rectal cancer was better than that of the clinical model in both the training and test sets.

The AUC value of the combined model was 0.74, which has a potentially greater advantage than that of the clinical model with an AUC value of 0.62 in the training dataset, demonstrating that combing clinical and radiomics features may improve the evaluation performance for LN metastases. When the clinical and clinical-radiomics combined models were applied to the testing cohort, the diagnostic performance was moderate with AUC values of 0.59 and 0.62, respectively, indicating that the combined model may demonstrate higher diagnostic efficiency for assessing LN status. A higher PPV was observed in the combined model than that in the clinical model, which may help select the high-risk patients with metastatic LNs. The detailed sensitivity, specificity, PPV, and NPV are shown in **Table 2**. The ROC analysis results are displayed in **Figure 3**.

**Table 2:** Comparison of results of the evaluation models in the training and testing cohorts.

	Models	SEN (%)	SPE (%)	PPV (%)	NPV (%)	AUC (95% CI)	*P* value
Training	Clinical model	48.3	72.9	40.0	79.0	0.62 (0.54-0.70)	< 0.001
	Combined model	48.8	88.0	84.3	57.6	0.74 (0.68-0.81)	
Testing	Clinical model	41.9	72.9	44.8	70.5	0.59 (0.47-0.71)	0.707
	Combined model	39.6	78.8	75.9	42.6	0.62 (0.49-0.74)	

SEN, sensitivity; SPE, specificity; PPV, positive predictive value; NPV, negative predictive value; AUC, area under the receiver operating characteristic curve; CI, confidence interval.

### Construction of the clinical-radiomics signature and its clinical utility

**Figure 4:**
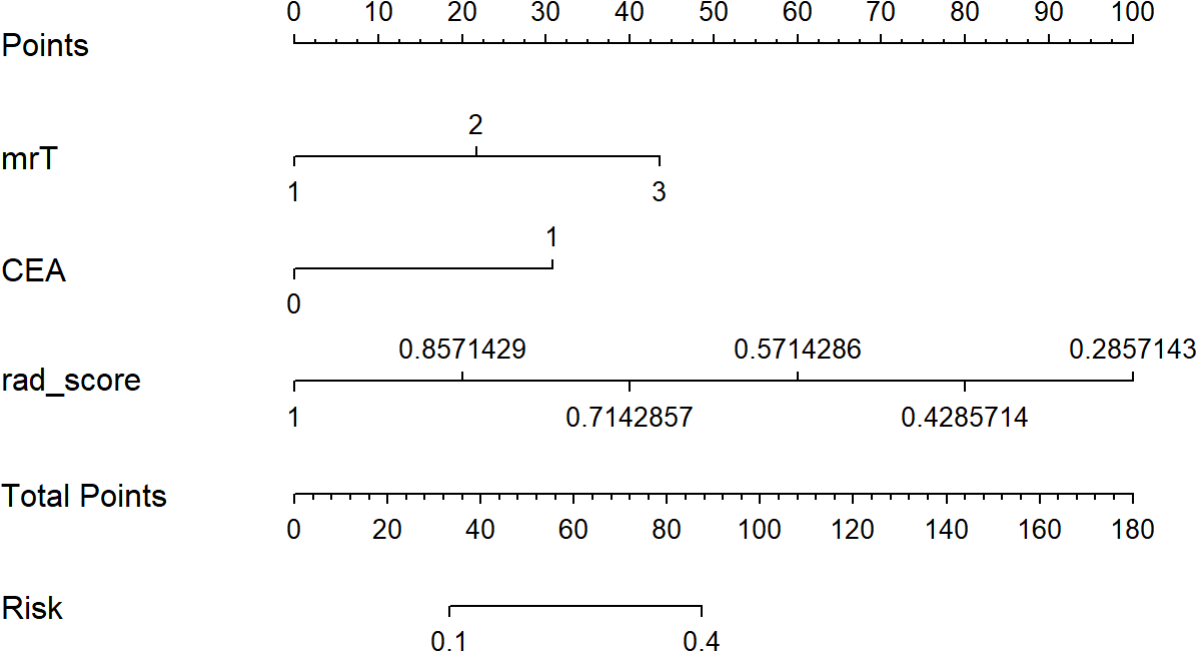
The developed clinical-radiomics nomogram for evaluating the probability of lymph node metastases.

**Figure 5:**
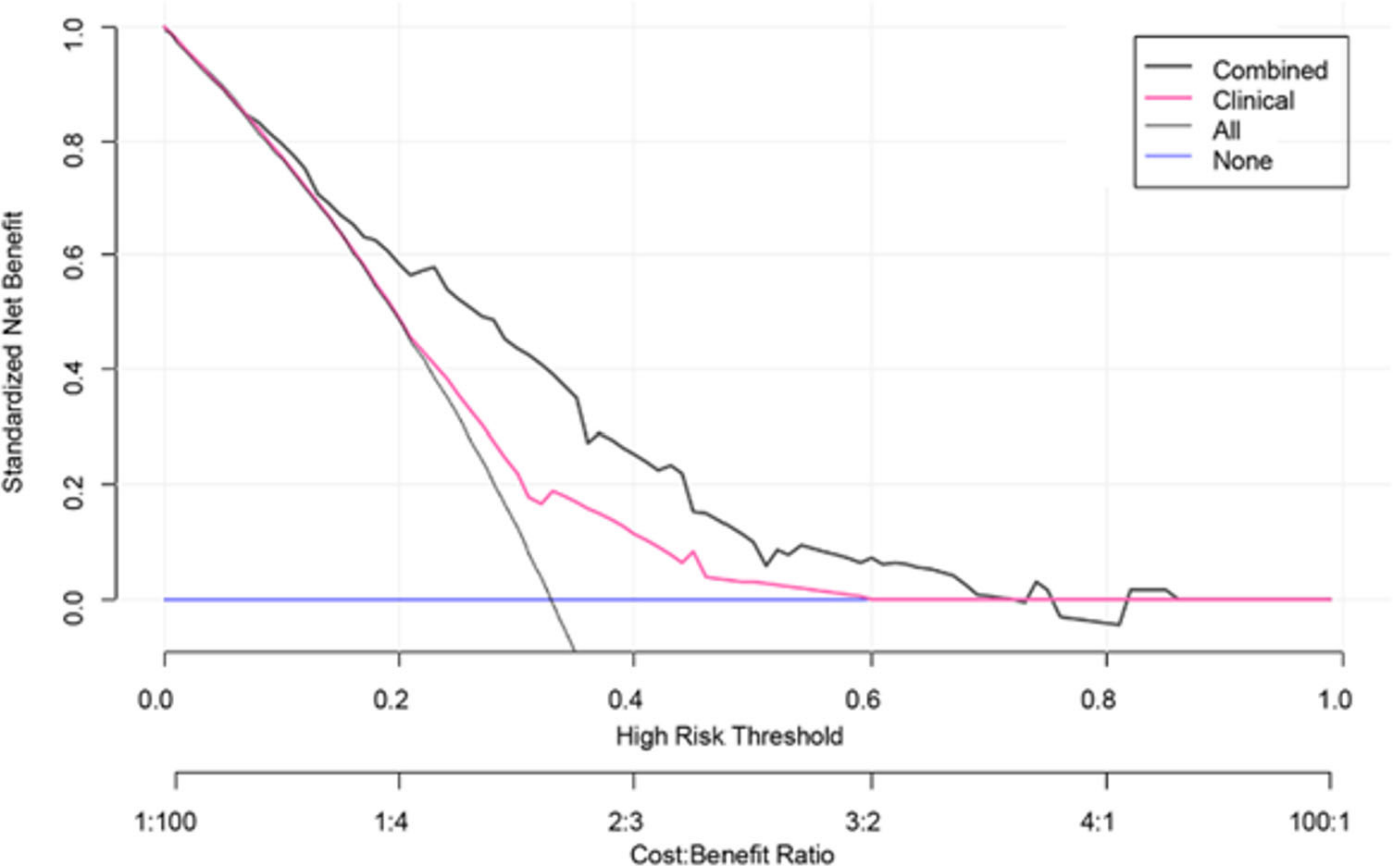
Decision curve analysis (DCA) for the clinical model and clinical-radiomics combined model. DCA indicated that when the threshold probability was between 0.1 and 0.7, using clinical-radiomics combined model to evaluate lymph node metastases gains more benefit than the clinical model.

A clinical-radiomics nomogram was exploited with the selected radiomics and clinical features (**Figure 4**). The DCA results are shown in **Figure 5**. The DCA demonstrated that the clinical-radiomics combined model to evaluate LN metastases was more beneficial than the clinical model when the threshold probability was between 0.1 and 0.7.

### Comparison of diagnostic efficiency between the combined model and radiologists

When comparing the performance of the combined model with 2 senior radiologists in the gastrointestinal imaging in the testing dataset, the results showed that the combined model was equivalent to reader 1 (AUC: 0.62) and slightly superior to reader 2 (AUC: 0.60). However, the PPV of 75.9% for combined model was higher than those of the 2 readers (54.9% for reader 1 and 56.3% for reader 2). Furthermore, compared with the relatively objective combined model, the interobserver agreement for 2 readers was not satisfactory with a kappa value of 0.416. The detailed results are demonstrated in **Table 3**.

**Table 3:** Comparison of the clinical-radiomics model and radiologists in the testing cohort.

Models	SEN (%)	SPE (%)	PPV (%)	NPV (%)	AUC (95% CI)	*P* value vs the combined model
Combined model	39.6	78.8	75.9	42.6	0.62 (0.49-0.74)	N/A
Senior radiologist 1	39.4	84.3	54.9	74.1	0.62 (0.55-0.69)	0.519
Senior radiologist 2	31.3	88.2	56.3	72.6	0.60 (0.53-0.67)	0.262

SEN, sensitivity; SPE, specificity; PPV, positive predictive value; NPV, negative predictive value; AUC, area under the receiver operating characteristic curve; CI, confidence interval.

## Discussion

In the present study, we explored the diagnostic value of the clinical-radiomics model to preoperatively evaluate LN metastasis in patients with low T-staging (mrT1-3a) rectal cancer. Our results demonstrated that the combined model incorporating radiomics features and the clinical factors could achieve a higher diagnostic efficiency for LN metastasis in the testing cohort, with an improved PPV of 75.9% from 44.8% for the clinical model, 54.9% for reader 1, and 56.3% for reader 2. The higher PPV indicated that the combined model could help identify truly high-risk patients with LN metastasis. Relatively high specificities of 88.0% and 78.8% for the combined model in the training and testing cohorts were also achieved, suggesting that the model was reliable and could eliminate more false-negative. Furthermore, we developed a clinical-radiomics nomogram as the preoperative individualized and visualized tool to provide the estimated probability of LN metastasis for a newly diagnosed low T-staging rectal cancer patient, which facilitates the tailored treatment. The nomogram could provide more objective model for LN status evaluation compared with the human assessment. DCA was also implemented to confirm the clinical benefit.

The accurate evaluation of LN metastasis based on observable MRI features in rectal cancer patients remains challenging. At present, MRI is the first choice for local TN staging of rectal cancer, however, the on-going evaluation accuracy and interobserver agreement of LN metastasis are not satisfactory (23). More than 25% of LNs are likely to be over-staged and unnecessary preoperative CRT will be performed, leading to possible complications and disease aggravation (24). On the contrary, if LNs are underestimated, receiving preoperative CRT not in time will affect the prognosis of patients. LN metastasis is a key indication and prognostic factor for preoperative CRT in rectal cancer patients with T1-3a staging, which has been emphasized in treatment guidelines for rectal cancer (4, 5). Therefore, accurate evaluation of LNs status is crucial for clinicians in making a personalized treatment plan for patients with low T-staging rectal cancer. However, it has been reported that about more than 50% of the involved LNs in rectal cancer are less than 5 mm in size (25). It is technically difficult to directly perform 3D segmentations on LNs. In this study, we evaluated primary tumor lesions to uncover the status of LNs, as some studies have indicated that the nature of the primary tumor is consistent with the metastatic LN (26, 27).

Previous studies have reported the diagnostic value of radiomics models in predicting LN metastasis in rectal cancer (17–19, 28–34). Huang et al. (30) developed a radiomics model based on enhanced CT to predict LN metastasis in CRC cancer patients and obtained an AUC value of 0.778. However, MRI is regarded as the most common method for local staging of rectal cancer. Several studies demonstrated that radiomics based on MRI had a better diagnostic performance in distinguishing LN status (31–33). However, most previous studies performed two-dimensional (2D) region of interest (ROI) on single-slice image at the level of the largest section of the primary tumor. 3D VOIs were constructed in our study, which can be more representative of the lesion heterogeneity than 2D ROIs (34). In addition, prior studies about the radiomics predicting LN metastasis focused on local advanced rectal cancer (17–19, 28, 29), few studies on radiomics have been conducted to identify LN metastasis in low T-staging rectal cancer. In this study, our results revealed that radiomics analysis could provide added value in evaluating LN metastasis for low T-staging rectal cancer, especially for the PPV and specificity, which could aid in the high-risk patients for LN metastasis in low T-staging rectal cancer. Additionally, the interobserver agreement of 2 senior radiologists for LN status assessment was moderate (kappa=0.416). The radiomics score in the clinical-radiomics nomogram was a relatively objective model, which could avoid the diagnosis inconsistency between different readers.

Radiomic features could reveal subtle changes in rectal tumor lesions that are difficult to identify with our naked eyes (35, 36). In this study, we selected 14 out of 960 radiomic features extracted from oblique axial T2WI images. These 14 radiomic features included 2 first-order features and 12 texture features. These radiomics features are also indices of the different growing patterns and texture that demonstrate subtle alternations of rectal cancer morphology and intratumor heterogeneity (37–39). Texture features account for the vast majority of radiomics features in our study. Our results were similar to prior studies, indicating that texture features are better than histogram-based or shape features in evaluating tumor prognosis (40, 41).

There are some limitations in our study. Firstly, the data used for radiomic feature extraction included only T2WI images since the T2WI sequences were most commonly used for local staging of rectal cancer. The value of DWI for improving diagnostic performance of radiomics models should be further explored. Secondly, the characteristics of primary tumor were used to evaluate LN metastasis. It is difficult to perform segmentation directly on LNs and radiological-pathological one-to-one matching of LNs. Thirdly, the sample size of LN metastasis in patients with T1-3a stage was relatively small. Investigations with a larger sample size will be required to confirm and improve the diagnostic performance. Fourthly, the mrT staging during the patient recruitment was based on the radiological reports. There may be some bias due to the different experience of radiologists. In addition, the cases are all from our institution, multicenter studies including different MRI scanners will be warranted to validate the generalization of the developed model.

## Conclusions

In conclusion, this study presented that T2WI-based radiomics combined with clinical data could facilitate non-invasively LN metastasis evaluation for the mrT1-3a staging rectal cancer, which may assist in making individual treatment strategy for rectal cancer patients.

## Data availability statement

The raw data supporting the conclusions of this article will be made available by the authors, without undue reservation.

## Ethics statement

The studies involving humans were approved by the Institutional Ethics Committee of Xinhua Hospital, Shanghai Jiao Tong University School of Medicine. The studies were conducted in accordance with the local legislation and institutional requirements. Written informed consent for participation was not required from the participants or the participants’ legal guardians/next of kin in accordance with the national legislation and institutional requirements.

## Author contributions

XD, GR and YC: Data curation, Methodology, Investigation, Formal analysis, Writing-original draft, Writing-review and editing, Visualization. DW and HL: Investigation, Writing-review and editing, Validation, Supervision, Visualization. HY: Investigation, Writing-review and editing, Validation, Supervision, Visualization. TZ: Data curation, Methodology, Investigation, Writing-review and editing. YG and SD: study design, Software, Investigation, Writing-review and editing, Validation, Supervision, Visualization. QY: Data curation, Investigation, Writing-review and editing. ZZ: Investigation, Writing-review and editing. SY: Writing-review and editing. All authors contributed to the article and approved the submitted version.
